# 2D Quantitative Structure-Property Relationship Study of Mycotoxins by Multiple Linear Regression and Support Vector Machine

**DOI:** 10.3390/ijms11093052

**Published:** 2010-08-31

**Authors:** Roya Khosrokhavar, Jahan Bakhsh Ghasemi, Fereshteh Shiri

**Affiliations:** 1 Food and Drug Laboratory Research Center, MOH & ME, Tehran, Iran; E-Mail: khosrokhavar_r@yahoo.com; 2 Department of Chemistry, Faculty of Sciences, K.N., Toosi University of Technology, Tehran 16617, Iran; 3 Faculty of Chemistry, Razi University, Kermanshah, Iran; E-Mail: fereshteh.shiri@gmail.com

**Keywords:** QSPR, mycotoxins, SVM, MLR, genetic algorithm, William’s Plot

## Abstract

In the present work, support vector machines (SVMs) and multiple linear regression (MLR) techniques were used for quantitative structure–property relationship (QSPR) studies of retention time (t_R_) in standardized liquid chromatography–UV–mass spectrometry of 67 mycotoxins (aflatoxins, trichothecenes, roquefortines and ochratoxins) based on molecular descriptors calculated from the optimized 3D structures. By applying missing value, zero and multicollinearity tests with a cutoff value of 0.95, and genetic algorithm method of variable selection, the most relevant descriptors were selected to build QSPR models. MLR and SVMs methods were employed to build QSPR models. The robustness of the QSPR models was characterized by the statistical validation and applicability domain (AD). The prediction results from the MLR and SVM models are in good agreement with the experimental values. The correlation and predictability measure by r^2^ and q^2^ are 0.931 and 0.932, repectively, for SVM and 0.923 and 0.915, respectively, for MLR. The applicability domain of the model was investigated using William’s plot. The effects of different descriptors on the retention times are described.

## 1. Introduction

Fungi are major plant and insect pathogens, but they are not nearly as important as agents of disease in vertebrates, *i.e.*, the number of medically important fungi is relatively low. Growth of fungi on animal hosts produces diseases collectively known as mycoses, while dietary, respiratory, dermal, and other exposures to toxic fungal metabolites produce diseases collectively called mycotoxicoses. Mycotoxicoses are examples of “poisoning by natural means” and thus are analogous to the pathologies caused by exposure to pesticides or heavy metal residues. The symptoms of mycotoxicosis depend on the type of mycotoxin; the amount and duration of the exposure; the age, health, and sex of the exposed individual; and many poorly understood synergistic effects involving genetics, dietary status, and interactions with other toxic insults. Thus, the severity of mycotoxin poisoning can be compounded by factors such as vitamin deficiency, caloric deprivation, alcohol abuse, and infectious disease status. In turn, mycotoxicoses can heighten vulnerability to microbial diseases, worsen the effects of malnutrition, and interact synergistically with other toxins [1].

Studies have shown that a number of mycotoxins have carcinogenic properties. Some of them are clearly DNA-reactive and for others DNA reactivity may not be the mode of action. When the endpoint is cancer, *in vitro* or *in vivo* studies may need to be designed to elucidate possible molecular events related to gene expression, modifications of relevant proto-oncogenes or tumor suppressor genes, and genomic instability, as this will help in gaining an understanding of the mode of action underlying the carcinogenic process and in the characterization of hazard. Mycotoxins may also cause developmental effects including birth defects, affect the reproductive system, affect the immune system, exhibit hormonal activity, affect specific target organs and may be neurotoxic. In addition to these diverse organ or site-specific actions, mycotoxins may affect the gastrointestinal system, cause skin irritation, have hematological effects and reduce growth [2–4].

Mycotoxins usually enter the body via ingestion of contaminated foods, but inhalation of toxigenic spores and direct dermal contact are also important routes. Mycotoxins occurring in food commodities are secondary metabolites of a range of filamentous fungi, which can contaminate food or food crops throughout the food chain. Although many hundreds of fungal toxins are known, a more limited number are generally considered to play an important part in food safety and for these a range of analytical methods have been developed [5].

Microfungi are a rich source of chemical diversity [6–8], and together with the actinomycetes they are the source of more than 50% of metabolites utilized by the pharmaceutical industry in either the native form or as derivatives [9–12]. As only a small part of mycota is known and most fungi produce several unknown metabolites, fungi are still one of the most promising microbiotic sources for new lead compounds. Therefore, developing theoretical models to predict the property (e.g., retention time) of mycotoxins is necessary as they toxicity is very important for humans and animals.

Since the chemical diversity is very high within the micro-fungi almost all types of chemical structure can be expected in an extract, e.g., small acids, alcohols, ketones, alkaloids, antraquinones and cyclic peptides. To cope with this broad range of chemical structures, most methods are based on reversed- phase liquid chromatography combined with diode array detection (DAD) and atmospheric ionization [electrospray ionization (ESI) and atmospheric pressure chemical ionization (APCI)] mass spectrometry (MS). Nearly all methods use water–acetonitrile gradient elution on reversed-phase C_18_ and C_8_ columns, although methods for very polar and highly ionized components, using perfusion chromatography and hydrophilic interaction chromatography have been described [13].

However, only a few reports have investigated the quantitative correlation between the molecular parameters and the property of retention time of mycotoxins [14]. The computational methods used to calculate/predict retention time can be classified into two categories. One approach is to use a mathematical equation to correlate retention time with the molecular parameters. The other methods are more empirically based on QSPR approaches using multiple linear regression (MLR) and support vector machine (SVM) techniques. Of those previous studies that aimed to predict the retention time, the most promising method has been to use the QSPR approach: QSPR methods have been successfully used to predict many physicochemical properties. The advantage of this approach over other methods lies in the fact that the descriptors used can be calculated from the structure alone and are not dependent on any experimental properties. Once the structure of a compound is known, any descriptor can be calculated, no matter whether it is found or not. This means that once a reliable model is established, we can use this method to predict properties of compounds. Therefore, quantitative structure- property relationship (QSPR) is a useful tool to predict the retention time, avoiding long and tedious separation optimization. QSPR studies can also tell us which of the structural factors may play an important role in the determination of retention time.

After the calculation of molecular descriptors, many different chemometrics methods, such as multiple linear regression (MLR), partial least squares regression (PLS), different types of artificial neural networks (ANN), genetic algorithms (GAs), and support vector machine (SVM) can be employed to derive correlation models between the molecular structures and properties. As a new and powerful modeling tool, support vector machine (SVM) has gained much interest in pattern recognition and function approximation applications recently. In bioinformatics, SVMs have been successfully used to solve classification and correlation problems. SVMs have also been applied in chemistry, for example, the prediction of retention index of protein [15], and other QSAR studies. Compared with traditional regression and neural networks methods, SVMs have some advantages, including global optimum, good generalization ability, simple implementation, few free parameters, and dimensional independence [16]. The flexibility in classification and ability to approximate continuous function make SVMs very suitable for QSAR and QSPR studies. In the present paper, we introduce the applications of support vector regression (SVR) for correlation problems in QSAR and compare its performance with MLR method.

## 2. Results and Discussion

54 descriptors were calculated by the ChemOffice software. By applying missing value, zeroand multicollinearity tests with a cutoff value of 0.95 and variable selection by genetic algorithm, the number of descriptors was reduced to 22. The stepwise regression routine was used to develop the linear model for the prediction of the retention time of mycotoxins using calculated structural descriptors. The best linear model contained four molecular descriptors. The regression coefficients of the descriptors, Mean effect and variable inflation factors (VIF) are listed in Table 1.

Positive values in the regression coefficients show that the indicated descriptors contribute positively to the value of t_R_, whereas negative values indicate that the greater the value of the descriptor, the lower the value of t_R_. In other words, increasing the electronic energy (ElcE), dipole length (DPLL)and Lowest Unoccupied Molecular Orbital energy (LUMO) will decrease t_R_, and the increase in the C log*P* increases the extent of t_R_ of the compounds.

With comparison of the mean effects of the descriptors appearing in MLR model, it is observed that the ElcE of the molecules has the largest effect on the t_R_ of the compound. The mean effect of a descriptor is the product of its mean and the regression coefficient in the MLR model [17].

Based on the variable inflation factor (VIF) values of the four descriptors shown in Table 1, it has been found that the descriptors used in the model have very low inter-correlation. Correlation between these descriptors and property as correlation matrix of measured data are given in Table 2. Correlation coefficients measure how closely two values (descriptor and property) are related to each other by a linear relationship. If a descriptor has a correlation coefficient of 1, it describes the property exactly. A correlation coefficient of zero means the descriptor has no relevance. It is seen that C logP is positivelycorrelated to the property with a correlation coefficient equal to 0.82126.

**Figure 1:**
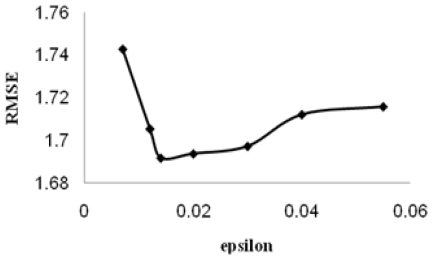
The selection of the optimal epsilon for SVM (C = 4).

**Figure 2:**
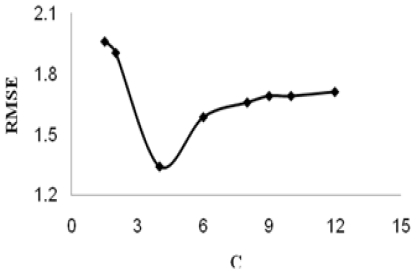
The selection of the optimal capacity factors for SVM (ɛ = 0.01).

After establishing models by MLR, the support vector machines were used to compare the performance of MLR based on the same subset of descriptors. Similar to other multivariate statistical models, the performances of SVM for regression depend on the combination of several parameters. They are capacity parameter *C*, *ɛ* of *ɛ*-insensitive loss function, the kernel type *K*, and its corresponding parameters. *C* is a regularization parameter that controls the tradeoff between maximizing the margin and minimizing the training error. If *C* is too small, then insufficient stress will be placed on fitting the training data. If *C* is too large, then the algorithm will overfit the training data. The linear kernel function was used for the SVR model in our study for investigation of the linear relationship between the theoretical molecular descriptors and the retention time. The optimal value for *ɛ* depends on the type of noise present in the data, which is usually unknown. Even if enough knowledge of the noise is available to select an optimal value for *ɛ*, there is the practical consideration of the number of resulting support vectors. *ɛ*-insensitivity prevents the entire training set meeting boundary conditions and so allows for the possibility of sparsity in the dual formulation’s solution. So, choosing the appropriate value of *ɛ* is critical from theory. To find an optimal ɛ, the root mean squares error (RMSE) on LOO cross-validation on different *ɛ* was calculated. The curve of RMSE *versus* the epsilon (*ɛ*) is shown in Figure 1. The optimal *ɛ* was found to be 0.014. The other important parameter is regularization parameter *C*, whose effect on the RMSEis shown in Figure 2. The optimal *C* was found to be 4.

**Figure 3:**
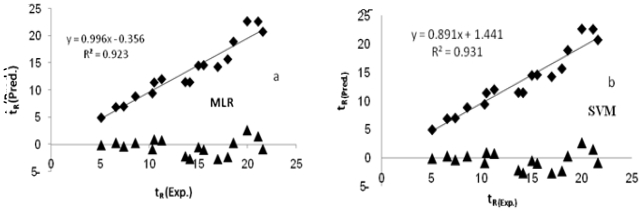
t_R_ estimated by MLR (top panel) and SVM (bottom panel) modeling *versus* experimental values and residual *versus* experimental t_R_.

Satisfied with the robustness of the QSPR model developed using the training set, we applied the QSPR model to an external data set of 17 mycotoxins comprising the test set. The predicted results are given in Table 3. The squared correlation coefficient between experimental and predicted t_R_ values for the test set for both models is significant. Figure 3 shows the quality of the fit. Also the random distribution of residuals about zero mean in Figure 3 confirms the good predictive ability of the models.

The statistical parameters calculated for the MLR and SVM models are represented in Table 4. In this table, statistical parameters root mean squared error of prediction (RMSEP), standard error of prediction (SEP), relative error of prediction (REP%) and the others parameters obtained by applying the MLR and SVM methods to the test set indicate a good external predictability of the QSPR models. The results also show that both MLR and SVM methods could model the relationship between t_R_ and their electronic and thermodynamic descriptors, while model using SVM based on these same sets of descriptors produced an even better model with a better predictive ability than the MLR model. SVM performs better on the whole due to embodying the structural risk minimization principle and the advantage over other techniques of converging to the global optimum and not to a local optimum.

### 2.1. Definition of the Applicability Domain of the Model

Once a QSPR model is obtained, another crucial problem is the definition of its applicability domain (AD). For any QSPR model, only the predictions for chemicals falling within its AD can be considered reliable and not model extrapolations. There are several methods for defining the AD of QSPR models [18], but the most common one is determining the leverage values for each compound [19]. To visualize the AD of a QSPR model, the plot of standardized residuals *versus* leverage values (*h*)(the William’s plot) was exploited in this study, which played a double role. Firstly, it described the impacts of the objects on models by the values of their leverages. Leverage indicates a compound’s distance from the centroid of *X*. The leverage of a compound in the original variable space is defined as [20]:

(1) $$h_{i}=x_{i}^{T}{\left(X^{T}X\right)}^{-1}x_{i}$$

where *xi* is the descriptor vector of the considered compound and *X* is the descriptor matrix derived from the training set descriptor values. The warning leverage (h*) is defined as [18]:

(2) $$h^{*}=\frac{3P}{n}$$

where *n* is the number of training compounds, *p* is the number of model variables plus one. The leverage (*h*) greater than the warning leverage (h^*^) suggested that the compound was very influential on the model. Secondly, it presented the Euclidean distances of the compounds to the model measured by the cross-validated standardized residuals. The cross-validated standardized residuals greater than three standard deviation (*s*) units classified the compound as a response outlier.

**Figure 4:**
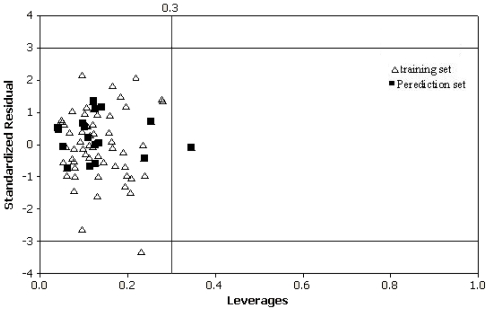
Williams plot of standardized residual *versus* leverage.

The Williams plot for the presented SVM model is shown in Figure 4. From this plot, the applicability domain is established inside a squared area within ±3 standard deviations and a leverage threshold h^*^ of 0.3. For making predictions, predicted t_R_ data must be considered reliable only for those compounds that fall within this AD on which the model was constructed. It can be seen from Figure 4 that the majority of compounds in the data set are inside this area. However, only one compound in prediction set(squares at 0.33 h) slightly exceeds the critical hat value that the developed SVM model has good generalizability and predictivity for the compound with descriptor values significantly far from the centroid of the descriptor space. Also, compound **2** in the training set is wrongly predicted (>3 s), but with lower leverage values (*h* < h^*^). These erroneous predictions could probably be attributed to wrong experimental data rather than to molecular structures [19].

### 2.2. Interpretation of Descriptors

By interpreting the descriptors in the regression model, it is possible to gain some insight into factors that are likely to govern the retention time of mycotoxins. In regard to this point that all the descriptors in the final model together attributethe same property or activity, each one of the descriptors or their related coefficient takes into account a definitive amount of variance within property. However it can be concluded that the interpretation of a combination set of the descriptors would be much better than considering the result of the single descriptors. Of the four descriptors, C logP is thermodynamic and LUMO, DPLL and ElcE are electronic descriptors.

The octanol/water partition coefficient (C logP) characterizes the effectiveness of hydrophobicity of the compounds. C logP values can be calculated from molecular structure by summation of fragment values, which captures the nature of the hydrophobic regions of the molecule separately from hydrophilic regions. In the other words, it can be estimated from hydrophobic contributions of the chemical groups present in complex molecules [21,22]. The fact that similar descriptors have been reported to correlate with partition coefficients of different compounds suggests that this correlation model has wider applications [23]. A positive value in the regression coefficient for C logp demonstrates that with the increase of C logp, the value of t_R_ increases as well. In reversed-phase chromatography, compounds with higher hydrophobicities would make stronger interactions with mobile phase, which lead to having larger t_R_ within the compounds.

The other descriptors (LUMO, DPLL and ElcE) are electronic and their regression coefficient is negative, it means that as they increase, t_R_ decreases. In particular, electronic parameters are considered important in the establishment of QSAR models and are helpful to quantify different types of intermolecular and intramolecular interactions, as these interactions are usually responsible for properties of chemical and biological systems [24]. Dipole length is the electric dipole moment divided by the elementary charge. Electric dipole is a vector quantity, which encodes displacement with respect to the centre of gravity of positive and negative charges in a molecule. Dipole length encodes information about the charge distribution in molecules and is important for modeling polar interactions. Large substituents decrease the DPLL valuem which is not desirable [25,26]. The ElcE descriptor has the largest effect on the t_R_ of the compounds. The ElcE is the total electronic energy given in electron volt at 0 °C [27]. Involvement of electronic factors suggests the occurrence of either charge transfer or dipolar interactions. The transfer of a pair of electrons from the HOMO to the LUMO is, by definition, a reaction between a Lewis acid and a Lewis base. Thus, the parameter LUMO is a measure of the ability of a molecule to interact with the π and *n*-electron pairs of the other molecules. The reduction in energy in molecular orbital is the driving force for chemical bond formation [28]. The negative sign of the corresponding regression coefficient between t_R_ and LUMO indicates that, t_R_ increase with decrease in the magnitude of LUMO index. The present results reinforce previous findings [29,30].

## 3. Experimental Section

### 3.1. Data Set

The data set for this investigation was extracted from a work reported by Nielsen *et al*. [13]. These data are listed in Table 5. It can be seen from the table that the data set is diverse, consisting of aflatoxins, trichothecenes, roquefortines and ochratoxins. This data set was randomly divided into two groups: training (calibration) and prediction (test) sets. The training and prediction sets consisted of 50 and 17 molecules, respectively. The values of t_R_ were used as the dependent variables.

### 3.2. Descriptor Generation and Reduction

The molecular structures of data set were sketched using the ChemDraw Ultra module of the CS ChemOffice 2005 molecular modeling software version 9, supplied by Cambridge Software Company. Each molecule was “cleaned up” and energy minimization was performed using Allinger’s MM2 force filed and further geometry optimization was done using semiempirical AM1 (Austin Model) Hamiltonian and PM3 methods by default on the 3D-structure of molecules. A total of 54 molecular descriptors of differing types based on 3D structures were calculated to describe compound structural diversity. The descriptors calculated accounts three important properties of the molecules: (a) thermodynamic, (b) electronic and (c) steric, as they represent the possible molecular interactions which determined the retention time of the studied molecules.

After the calculation of molecular descriptors, any parameter which is not calculated (missing value) for any number of the compounds in the data set is rejected in the first step. Some of the descriptors were rejected because they contained a value of zero for all the compounds and have been removed (zero tests). In order to minimize the effect of colinearity and to avoid redundancy, we used amulticollinearity test with a cutoff value of 0.95, and subsequently discarded 10 parameters. Finally, a total set of 44 remaining descriptors were achieved and used to select the optimal subset of descriptors that have a significant contribution to the t_R_ property.

### 3.3. Descriptor Selection and Model Building

The basic strategy of QSPR analysis is to find optimum quantitative relationships between the molecular descriptors and desired property, which can then be used for the prediction of the property from only molecular structures. One of the most important problems involved in QSPR studies is to select optimal subset of descriptors that have significant contribution to the desired property. The well-known genetic algorithm is just a well-accepted method for solving this kind of problems.

After correlation analysis of the descriptors, we used MLR analysis on the molecular descriptors that resulted in genetic algorithm (GA) variable selection procedure. The GA-algorithm applied in this paper uses a binary representation as the coding technique for the given problem; the presence or absence of a descriptor in a chromosome is coded by 1 or 0. The GA performs its optimization by variation and selection via the evaluation of the fitness function (RMSECV). The algorithm used in this paper is an evolution of the algorithm described in Ref. [31], whose parameters are reported in Table 6. In our study, a genetic algorithm procedure was used for selection of descriptors using the PLS Toolbox (version 2.0, Eigenvector Company, USA). The GA is implemented in MATLAB (version 7.1, MathWorks, Inc.). By performing GA, 22 descriptors were retained for next analysis step.

Finally, descriptor-screening methods were used to select the most relevant descriptor to establish the models for prediction of the molecular property. Here, the stepwise regression method was used to choose the subset of the molecular descriptors.

After the descriptor was selected, multiple linear regression (MLR)[32] was used to develop the linear model of the property of interest, which takes the form below:

(3) $$y=b_{0}+b_{1}x_{1}+b_{2}x_{2}+\cdots +b_{n}x_{n}$$

In this equation, *y* is the property, that is, the dependent variable, *x*_1_−*x_n_* represent the specific descriptor, while *b*_1_− *b_n_* represent the coefficients of those descriptors, and *b_0_* is the intercept of the equation. The statistical evaluation of the data was obtained by the software SPSS. The SPSS software, (SPSS Ver. 11.5, SPSS Inc.), performed MLR analysis and variable selection by using stepwise method for the variable selection and modeling.

### 3.4. Theory of SVM

The foundation of support vector machines (SVM) has been developed by Vapnik, and they are gaining popularity due to many attractive features and promising empirical performance [33]. The formulation embodies the structural risk minimization (SRM) principle [32,33], which has been shown to be superior to the traditional empirical risk minimization (ERM) principle, employed by conventional neural networks. SRM minimizes an upper bound on VC dimension (“generalization error”), as opposed to ERM that minimizes the error on the training data. It is the difference that equips SVM with good generalization performance, which is the goal in statistical learning. Originally, SVM were developed for classification problems [34], and now, with the introduction of *ɛ*-insensitive loss function, SVM have been extended to solve nonlinear regression estimation [36].

Compared to other neural network regressors, there are three distinct characteristics when SVM are used to estimate the regression function. First of all, SVM estimate the regression using a set of linear functions that are defined in a high dimensional space. Second, SVM carry out the regression estimation by risk minimization where the risk is measured using Vapnik’s *ɛ*-insensitive loss function. Third, SVM use a risk function consisting of the empirical error and a regularization term which is derived from the SRM principle.

In support vector regression (SVR), the basic idea is to map the data *x* into a higher-dimensional feature space *F* via a nonlinear mapping Φ, and then to do linear regression in this space. Therefore, regression approximation addresses the problem of estimating a function based on a given data set G = {(*x_i_*,*d_i_*)}*i^n^* (*x_i_* is the input vector, *d_i_* is the desired value, and *n* is the total number of data patterns). SVM approximate the function using the following

(4) y=f(x)=wΦ(x)+b

where Φ(*x*) denotes the element wise mapping from *x* into feature space. The coefficients *w* and *b* are estimated by minimizing

(5) $$R_{SVMs}(C)=C\frac{1}{n}\sum_{i=1}^{n}L_{e}(d_{i},y_{i})+\frac{1}{2}{||w||}^{2}$$

(6) $$L_{ɛ}(d,y)=\{\{\begin{array}{ll} |d-y|- & ɛ|d-y|\geq ɛ\\ 0 & otherwise \end{array}$$

In Equation 5,*R_SVMs_* is the regularized risk function, and the first term $C\frac{1}{n}\sum_{i=1}^{n}L_{e}(d_{i},y_{i})$ is the empirical error (risk). They are measured by the *ɛ*-insensitiveloss function (*L_ɛ_*) given by Equation 6. This loss function provides the advantage of enabling one to use sparse data points to represent the decision function given by Equation 4. The second term $\frac{1}{2}||w|{|}^{2}$, on the other hand, is the regularization term. *C* is referred to as the regularized constant, and it determines the tradeoff between the empirical risk and the regularization term. Increasing the value of *C* will result in the relative importance of the empirical risk with respect to the regularization term to grow.

*ɛ* is called the tube size, and it is equivalent to the approximation accuracy placed on the training data points. Both *C* and *ɛ* are user-prescribed parameters.

Finally, by introducing Lagrange multipliers (*a_i_*, a_i_^*^) andexploiting the optimality constraints, the decision functiongiven by Equation 4 has the following explicit form:

(7) $$f(x,a_{i},a_{i}^{*})=\sum (a_{i}-a_{i}^{*})K(x,x_{i})+b$$

Based on the Karush-Kuhn-Tucker (KKT) conditions of quadratic programming, only a number of coefficients (*a_i_*, a_i_^*^) will assume nonzero values, and the data points associated with them could be referred to as support vectors. In Equation 7, the kernel function *K* corresponds to *K*(*x*, *x_i_*) = Φ(*x*).Φ(*x_i_*). One has several possibilities for the choice of this kernel function, including linear, polynomial, splines, and radial basis function. The elegance of using the kernel function lies in the fact that one can deal with feature spaces of arbitrary dimensionality without having to compute the map Φ(*x*) explicitly.

The overall performances of SVM models were evaluated in terms of root mean square error (RMSE), which was defined as below:

(8) RMSE=∑i=1ns(yk-y^k)2nS

where *y_k_* is the desired output, *ŷ_k_* is the predicted value and *n*s is the number of samples in the analyzed set.

The predictive power of the models developed on the calculated statistical parameters standard error of prediction (SEP) and relative error of prediction (REP %) as follows:

(9) $$SEP={\left[\frac{\sum_{i=1}^{n}{\left({\tilde{y}}_{i}-y_{i}\right)}^{2}}{n-1}\right]}^{0.5}$$

(10) $$REP(\%)=\frac{100}{\bar{y}}{\left[\frac{1}{n}\sum_{i=1}^{n}{\left({\tilde{y}}_{i}-y_{i}\right)}^{2}\right]}^{0.5}$$

where *ỹ_i_*, *y_i_* and *ȳ* are the predicted, experimental and mean activity property, respectively.

All calculations in this work were carried out by using Matlab (V 7.1, The Mathworks, Inc.) and the SVM toolbox developed by Gunn [37].

### 3.5. Validation Test

The main goal in QSPR studies is to obtain a model with the highest predictive ability. In order to evaluate the predictive ability of our QSPR model, we used the method described by Golbraikh and Tropsha [38] and Roy and Roy [39]. The determination coefficient in prediction (*q^2^_test_*) was calculated using the following equation [39]:

(11) $$q_{test}^{2}=1-\frac{\sum {\left(y_{pred_{test}}-y_{test}\right)}^{2}}{\sum {\left(y_{test}-\bar{y}\right)}^{2}}$$

where *y_predtest_* and *y_Test_* are the predicted values based on the QSPR equation (model response) and experimental activity values, respectively, of the external test set compounds. *ȳ* is the mean activity value of the training set compounds. Further evaluation of the predictive ability of the QSAR model for the external test set compounds was done by determining the value of *r_m_*^2^ using the following equation [39]:

(12) $$r_{m}^{2}=r^{2}(1-|\sqrt{r^{2}-r_{0}^{2}}|)$$

where *r^2^* is the squared Pearson correlation coefficient for regression calculated using *Y* = *a + bx*; “*a”* is referred to as the *y*-intercept, “*b*” is the slope value of regression line, and *r*_0_^2^ is the squared correlation coefficient for regression without using y-intercept and the regression equation was *y* = *bx.* Both *r_2_* and *r*_0_^2^ between experimental and predicted values for the external test set compounds were calculated using the regression of analysis Toolpak option of Excel. If *r_m_*^2^ value for a give model is >0.5, it indicates the good external predictability of the developed model.

The values of *k* and *k*′, slopes of the regression line of the predicted property *versus* actual property and *vice versa*, were calculated using the following equations [38]:

(13) $$k=\frac{\sum y_{i}{\tilde{y}}_{i}}{\sum {\tilde{y}}_{i}^{2}}k^{\prime }=\frac{\sum y_{i}{\tilde{y}}_{i}}{\sum y_{i}^{2}}$$

where *ỹ_i_* and *y_i_* are the predicted and experimental property, respectively. The values of *k* and *k*′ are within the specified range of 0.85 and 1.15 [36]. The value of [r^2^ − r_0_^2^/r^2^] and [r^2^ − r_0_^2^′/r^2^] are less than 0.1 (stipulated value)[38]. *r*_0_^2^ and *r*_0_^2^′ are correlation coefficient of regression between the predicted and experimental property of compounds in the test set and *vice versa* without using y-intercept.

To further check the inter-correlation of descriptors variance inflation factor (VIF) analysis was performed. The VIF value is calculated from 1/1 − r^2^, where *r*^2^ is the multiplecorrelation coefficient of one descriptor’s effect regressed on the remaining molecular descriptors. If the VIF value is larger than 10, information of the descriptor could be hidden by correlation of descriptors [40].

## 4. Conclusions

In recent years, attention has been paid to QSAR/QSPR methods as an interesting complement, or even as an expensive, time consuming alternative, to laboratory data. In this paper, new QSPR models have been developed for predicting the t_R_ of a diverse set of mycotoxins from the molecular structure alone. We have compared two linear models, MLR and SVM, with the data set. The obtained results show that both MLR and SVM methods could model the relationship between t_R_ and their electronic and thermodynamic descriptors; on the same sets of descriptors, using SVM based produced a better model with a better predictive ability than the MLR model. SVM exhibit the better overall performance due to embodying the structural risk minimization principle and some advantages over the other techniques of converging to the global optimum and not to a local optimum. By performing model validation, it can be concluded that the presented model is a valid model and can be effectively used to predict the t_R_ of mycotoxins with an accuracy approximating the accuracy of experimental t_R_ determination. Moreover, the mechanism of the model was interpreted, and the applicability domain of the model was defined. It can be reasonably concluded that the proposed model would be expected to predict t_R_ for new organic compounds or for other organic compounds for which experimental values are unknown. Additionally, the presented method could also identify and provide some insight into what structural features are related to the t_R_ property of organic compounds.

## Figures and Tables

**Table 1:** Details of the constructed QSPR model.

Descriptor	Coefficient	Mean effect	VIF^e^
C logP^a^	2.6951(±0.2248)	5	1.006
ElcE^b^	−0.0002(±0.0001)	8	1.246
DPLL^c^	−1.091(±0.2981)	−3.875	1.556
LUMO^d^	−1.6922(±0.5521)	0.594	1.287
Constant	3.1912(±1.7569)	_	_

a = The octanol/water partition coefficient

b = Electronic energy

c = Dipole length

d = Lowest Unoccupied Molecular Orbital energy

e = Variable inflation factors

**Table 2:** Correlation matrix for MLR model.

	t_R_	C logP	ElcE	DPLL	LUMO
t_R_	1				
C logP	0.821263	1			
ElcE	−0.21234	0.05977	1		
DPLL	−0.07144	0.004813	−0.32903	*1*	
LUMO	−0.12041	−0.05044	0.000773	−0.45025	1

**Table 3:** Comparison of experimental and predicted values of t_R_ for prediction set by MLR and SVM models.

No.	Exp. ( t_R_)	MLR model		SVM model	

		Pred. (t_R_)	RE (%)	Pred. (t_R_)	RE (%)
21	5.1	4.97	2.55	5.03	1.37
4	6.6	6.91	−4.7	7.99	−21.06
23	7.4	7.03	5	8.35	−12.84
41	8.59	8.88	−3.38	10.08	−17.35
3	10.33	9.44	8.62	10.25	0.77
38	10.51	11.43	−8.75	12	−14.18
24	11.28	12.03	−6.65	12.37	−9.66
27	13.69	11.51	15.92	11.74	14.24
34	14.15	11.48	18.87	12.53	11.45
13	15.03	14.52	3.39	15.18	−1
25	15.56	14.61	6.11	14.79	4.95
37	17	14.29	15.94	15.08	11.29
11	18.02	15.7	12.87	16.37	9.16
46	18.6	18.91	−1.67	19.39	−4.25
65	20	22.66	−13.3	22.11	−10.55
29	21.12	22.61	−7.05	20.43	3.27
55	21.6	20.74	3.98	19.84	8.15

**Table 4:** The statistical parameters obtained by applying the MLR and SVM methods to the prediction set.

Parameters	MLR	SVM
RMSEP	1.504	1.341
REP^a^ (%)	10.902	9.719
SEP^b^	1.551	1.382
q^2^	0.915	0.932
R^2^	0.923	0.931
(R^2^-R_0_^2^)/R^2^	0.001	0.0118
(R^2^-R′_0_^2^)/R^2^	0.0108	0.0011
r_m_^2^	0.894	0.833
k	0.996	0.891
k′	0.926	1.045
NDS^c^	4	4

a = Relative error of prediction.

b = Standard error of prediction.

c = Number of descriptors.

**Table 5:** Experimental retention time (t_R_) of 67 compounds.

NO.	Compound	t_R_(min)	NO.	Compound	t_R_(min)
**Aflatoxins and their precursors**					
1	Aflatoxicol I	12.45	9	Austocystin A	21.57
2	Aflatoxin B_1_	11.50	10	Averufin	25.65
3	Aflatoxin B_2_	10.33	11	5-Methoxysterigmatocystin	18.02
4	Aflatoxin B_2_ α	6.60	12	Dihydroxysterigmatocystin	17.70
5	Aflatoxin G_1_	10.16	13	Methoxysterigmatocystin	15.03
6	Aflatoxin G_2_	8.97	14	Sterigmatocystin	18.91
7	Aflatoxin G_2_α	5.00	15	Norsolorinic acid	31.08
8	Aflatoxin M_1_	7.21	16	Parasiticol	10.73
**Trichothecenes**					
17	Nivalenol	1.27	27	HT-2 Toxin	13.69
18	Fusarenone X	2.35	28	T-2 Toxin	17.06
19	Deoxynivalenol	1.54	29	Acetyl-T-2 toxin	21.12
20	3-Acetyldeoxynivalenol	5.21	30	Trichodermin	16.13
21	15-*O*-Acetyl-4- deoxynivalenol	5.10	31	Trichodermol	9.69
22	Scirpentriol	1.82	32	7-α-Hydroxytrichodermol	2.59
23	15-Acetoxyscirpenol	7.40	33	Verrucarol	2.89
24	Diacetoxyscirpenol	11.28	34	4,15-Diacetylverrucarol	14.15
25	3α-Acetyldiacetoxyscirpenol	15.56	35	Trichothecin	16.29
26	Neosolaniol	3.19	36	Trichothecolone	3.63
37	Trichoverrol A	10.16			
**Roquefortines, ergot amines and related alkaloids**					
38	Agroclavine-I	17.00	51	Ergotamin	19.60
39	Auranthine	10.51	52	Fumigaclavine C	21.40
40	Aurantiamine	10.49	53	Marcfortine A	19.59
41	Aurantioclavine	14.30	54	Marcfortine B	17.39
42	Chanoclavine-I	8.59	55	Meleagrin	18.90
43	Costaclavine	17.00	56	Oxalin	21.60
44	Cyclopenin	11.60	57	Pyroclavine	14.81
45	Cyclopenol	6.20	58	Roquefortine C	20.50
46	Cyclopeptin	12.05	59	Roquefortine D	6.09
47	Dihydroergotamin	18.60	60	Rugulovasine A and B	8.43
48	Elymoclavine	5.34	61	Secoclavine	20.40
49	Epoxyagroclavine-I	10.00	62	α-Ergocryptin	19.20
50	Ergocristine	25.10			
**Ochratoxins**					
63	Ochratoxin α	5.60	66	Ochratoxin B-ethyl ester	19.41
64	Ochratoxin A-methyl ester	22.49	67	Ochratoxin α-methyl ester	16.16
65	Ochratoxin B-methyl ester	20.00			

**Table 6:** Parameters of genetic algorithm (GA).

Cross-Validation	Random subset
Number of subsets	4
Population size	64
Mutation rate	0.005
Window width	2
Initial term%	20%
Maximum generation	100
Convergence (%)	50
Cross-over	Double
